# *STK11* rs12977689 C>A gene polymorphism as a risk factor for coronary artery disease in type 2 diabetes patients

**DOI:** 10.7555/JBR.39.20250136

**Published:** 2026-05-21

**Authors:** Made Edwin Sridana, Wira Gotera, Bagus Ari, Pradnyana Dwi, Bagus Ari Pradnyana Dwi Sutanagera, Made Ratna Saraswati

**Affiliations:** 1 Internal medicine department, Faculty of Medicine, Universitas Udayana/Ngoerah hospital, Denpasar, Bali, Indonesia; 2 Cardiology department, Faculty of Medicine, Universitas Udayana/Ngoerah hospital, Denpasar, Bali, Indonesia

Dear Editor,

Macrovascular and microvascular diseases are the most frequent complications of type 2 diabetes mellitus (T2DM), with coronary artery disease (CAD) being particularly noteworthy^[1]^. Both environmental factors, such as dietary habits and lifestyle, and genetic factors contribute to the risk of CAD^[2]^. Notably, single-nucleotide polymorphisms (SNPs) in the serine/threonine kinase 11 (*STK11*) gene have been linked to the development of CAD in patients with T2DM through various mechanisms involving the adiponectin pathway. The activation of AMP-activated protein kinase (AMPK), which depends on STK11, can suppress IKK-NF-κB signaling, thereby reducing the production of proinflammatory cytokines and adhesion molecules, as well as interleukin-18-mediated endothelial cell death^[3–4]^. Adiponectin-stimulated STK11-AMPK signaling leads to increased activity of endothelial nitric oxide synthase (eNOS), which in turn boosts nitric oxide production. This may result in enhanced vasodilation, inhibition of platelet aggregation, and suppression of vascular smooth muscle proliferation^[5]^. Additionally, STK11-mediated AMPK phosphorylation inactivates acetyl-CoA carboxylase, leading to increased fatty acid oxidation. Overall, STK11 has been shown to act as an AMPK activator, contributing to many of AMPK's cellular effects, and thus plays a crucial anti-atherosclerotic role in coronary health^[4–7]^.

Despite the role of STK11 in regulating the cardiovascular system, there are limited studies on *STK11* and its genetic variants in the context of cardiovascular disease, particularly CAD. Several *STK11* gene polymorphisms exist, and a recent study in China found that the rs12977689 and rs35369365 polymorphisms were associated with CAD in patients with T2DM, showing particularly significant associations for rs12977689^[4]^. Based on allele frequency data, the A allele of *STK11* rs12977689 is present in approximately 33.6% of East Asian populations, 19.7% in Europeans, 14.3% in Latino/Admixed American populations, and 5.3% in African/African-American groups^[8]^.

Research on the *STK11* gene polymorphism and its association with the development of CAD in T2DM patients in Indonesia has not yet been conducted. Therefore, the present study aimed to investigate whether the *STK11* rs12977689 C>A polymorphism is a risk factor for CAD in patients with T2DM in Indonesia.

The present study was an analytical case-control study conducted at Ngoerah Hospital in Denpasar, Bali, Indonesia, from June to December 2022. Data were collected from primary assessments and medical records. Clinical variables included age, sex, smoking status, hypertension, lipid profile, duration of type 2 diabetes mellitus, and coronary artery disease diagnosis. In addition, peripheral venous blood samples were obtained from each participant for genomic DNA extraction. Genotyping of the STK11 rs12977689 polymorphism was performed using polymerase chain reaction (PCR)-based methods. The sampling method used was consecutive sampling according to inclusion and exclusion criteria. Inclusion criteria were patients diagnosed with T2DM based on clinical manifestations and laboratory findings at Ngoerah Hospital, with CAD patients allocated to the case group and non-CAD patients to the control group. Exclusion criteria encompassed patients with active infections, cancer, tumors, or autoimmune diseases.

The sample size was calculated using a significance level of 5% (*α* = 0.05) and a power of 80%. Based on an effect size of 0.37, the required sample size per group was 50, resulting in a total of 100 participants (50 controls and 50 cases) for the study. The effect size of 0.37 was chosen for the sample size calculation in accordance with general recommendations for medium-sized effects in genetic association studies. This effect size aligns with values commonly reported in previous studies exploring genetic variants associated with coronary artery disease in patients with type 2 diabetes.

The diagnosis of T2DM was based on the American Diabetes Association (ADA)/International Diabetes Federation (IDF) criteria. CAD was defined as the presence of coronary artery stenosis > 50% in at least one major coronary artery, confirmed by coronary angiography or spiral computed tomography coronary angiography. Non-CAD patients were selected based on clinical examinations and diagnostic tests to ensure the absence of significant CAD (stenosis ≤ 50%). Data regarding the duration of T2DM, age, sex, and other chronic illnesses were collected. Data on lipid profile, fasting blood glucose (FPG), and glycated hemoglobin (HbA1c) were also collected. Patients were treated according to standard protocols for diabetes management, which included insulin therapy and/or oral hypoglycemic agents. Genotyping of *STK11* rs12977689 was performed using conventional PCR followed by sequencing. This study was approved by the Institutional Review Board of Universitas Udayana/Ngoerah Hospital (Approval No. 1035/UN14.2.2.VII.14/LT/2022), and all participants provided informed consent.

Univariate analysis was performed to describe baseline characteristics. Variables with *P* < 0.25 in bivariate analysis were included in multivariate logistic regression to control for confounding factors and determine independent associations. All statistical analyses were performed using SPSS (version 27; IBM Corp., Armonk, NY, USA).

Baseline characteristics of the study subjects are presented in ***Table 1***. Males predominated in both the case and control groups, accounting for 84% and 78% of participants, respectively. The average ages of participants in the case group (61.10 ± 8.64 years) and control group (58.20 ± 9.93 years) were similar. In the case group, 33 participants (66.0%) carried the variant genotypes (CA/AA), while 17 participants (34.0%) had the wild-type CC genotype. In the control group, 22 participants (44.0%) carried variant genotypes, and 28 participants (56.0%) had the wild-type CC genotype. Comparative analysis revealed no significant difference in metabolic parameters, including fasting plasma glucose, low-density lipoprotein (LDL), total cholesterol, triglycerides, and HDL, between the groups. The body mass index also did not differ significantly between the groups. The duration of T2DM in the case group (5.78 ± 6.81 years) was comparable to that in the control group (6.85 ± 7.23 years). Additionally, there were no significant differences in the history of hypertension and smoking history between the groups.

**Table 1:** Baseline characteristics and analysis of risk factors for CAD in T2DM patients

Characteristics	Case (*n* = 50)	Control (*n* = 50)	*P*-value	OR	95% CI
Age (years, mean ± SD)	61.10 ± 8.64	58.20 ± 9.93	0.824	–	–
≥ 65 [*n* (%)]	11 (22.0)	18 (36.0)		1.220	0.509–2.925
< 65 [*n* (%)]	39 (78.0)	32 (64.0)		Ref	–
Sex [*n* (%)]			0.610		
Male	42 (84.0)	39 (78.0)		1.481	0.540–4.064
Female	8 (16.0)	11 (22.0)		Ref	–
Smoking history [*n* (%)]			0.068		
Yes	16 (32.0)	26 (52.0)		2.302	1.021–5.190
No	34 (68.0)	24 (48.0)		Ref	–
Hypertension [*n* (%)]			0.838		
Yes	31 (62.0)	29 (58.0)		1.181	0.530–2.632
No	19 (38.0)	21 (42.0)		Ref	–
T2DM duration (years, mean ± SD)	5.78 ± 6.81	6.85 ± 7.23	1.000	–	–
≥ 5 [*n* (%)]	27 (54.0)	27 (54.0)		1.000	0.455–2.196
< 5 [*n* (%)]	23 (46.0)	23 (46.0)		Ref	–
HbA1c (%, mean ± SD)	8.43 ± 2.22	7.93 ± 1.05	0.026^*^	–	–
≥ 7.5 [*n* (%)]	40 (80.0)	47 (94.0)		2.739	1.204–6.230
< 7.5 [*n* (%)]	10 (20.0)	3 (6.0)		Ref	–
FPG (mg/dL, mean ± SD)	146.52 ± 66.86	158.68 ± 77.98	0.424	–	–
≥ 130 [*n* (%)]	22 (44.0)	27 (54.0)		1.494	0.679–3.286
< 130 [*n* (%)]	28 (56.0)	23 (46.0)		Ref	–
Total cholesterol (mg/dL, mean ± SD)	160.50 ± 55.03	158.22 ± 57.66	0.715	–	–
≥ 240 [*n* (%)]	5 (10.0)	3 (6.0)		1.741	0.393–7.713
< 240 [*n* (%)]	45 (90.0)	47 (94.0)		Ref	–
Triglycerides (mg/dL, mean ± SD)	152.48 ± 108.19	143.62 ± 68.01	1.000	–	–
≥ 200 [*n* (%)]	10 (20.0)	10 (20.0)		1.000	0.375–2.664
< 200 [*n* (%)]	40 (80.0)	40 (80.0)		Ref	–
HDL (mg/dL, mean ± SD)	39.14 ± 10.45	48.22 ± 37.46	0.141	–	–
Low HDL [*n* (%)]	43 (86.0)	36 (72.0)		2.389	0.870–6.556
Normal HDL [*n* (%)]	7 (14.0)	14 (28.0)		Ref	–
LDL (mg/dL, mean ± SD)	98.88 ± 43.23	84.04 ± 55.44	0.839	–	–
≥ 70 [*n* (%)]	37 (74.0)	25 (50.0)		1.179	0.523–2.610
< 70 [*n* (%)]	13 (26.0)	25 (50.0)		Ref	–
BMI (kg/m^2^, mean ± SD)	24.09 ± 3.50	23.20 ± 3.94	0.317	–	–
≥ 25 [*n* (%)]	12 (24.0)	8 (16.0)		1.658	0.612–4.491
< 25 [*n* (%)]	38 (76.0)	42 (84.0)		Ref	–
*STK11* polymorphism			0.044^*^		
Polymorphism (CA/AA) [*n* (%)]	33 (66.0)	22 (44.0)		2.471	1.100–5.547
Wild type (CC) [*n* (%)]	17 (34.0)	28 (56.0)		Ref	–
^*^P < 0.05. Low HDL was defined as < 40 mg/dL in males and < 50 mg/dL in females; boundary values were included in the normal HDL category. Abbreviations: BMI, body mass index; HbA1c, hemoglobin A1c; FPG, fasting plasma glucose; HDL, high-density lipoprotein; LDL, low-density lipoprotein; T2DM, type 2 diabetes mellitus.					

As shown in ***Table 1***, bivariate analysis revealed a significant association between the *STK11* rs12977689 C>A polymorphism and CAD status (odds ratio [OR]: 2.471; 95% confidence interval [CI]: 1.100–5.547, *P* = 0.044), indicating that carriers of the polymorphic genotype had a 2.471-fold increased risk of CAD among T2DM patients. Bivariate analysis also examined associations between CAD status and several confounding variables, including age (*P* = 0.824), sex (*P* = 0.610), HbA1c (*P* = 0.026), smoking history (*P* = 0.068), hypertension history (*P* = 0.838), body mass index (BMI, *P* = 0.317), total cholesterol (*P* = 0.715), triglycerides (*P* = 1.000), LDL (*P* = 0.839), HDL (*P* = 0.141), duration of diabetes (*P* = 1.000), and FPG (*P* = 0.424).

Multivariate logistic regression analysis (***Table 2***) demonstrated that the *STK11* rs12977689 C>A polymorphism and HbA1c were significantly associated with CAD status in T2DM patients after adjusting for confounding factors. Specifically, carriers of the *STK11* rs12977689 genotype C>A had an increased risk of CAD by 2.389 fold, while each unit increase in HbA1c levels was associated with an elevated risk of CAD by 2.657 fold.

**Table 2:** Multivariate analysis with logistic regression

Variables	*B*	*SE*	Adjusted OR	CI	*P*
Polymorphism	0.849	0.450	2.389	1.041–5.487	0.040^*^
HbA1c	1.130	0.464	2.657	1.147–6.145	0.023^*^
Constant	−0.797	0.335	0.450		0.017^*^
^*^P < 0.05.Abbreviations: CI: confidence interval; HbA1c, hemoglobin A1c; OR, odds ratio.					

The present study demonstrated a significant association between the *STK11* rs12977689 C>A polymorphism and CAD risk in T2DM patients in both bivariate (OR = 2.471, 95% CI: 1.100–5.547, *P* = 0.044) and multivariate analyses (OR = 2.389, 95% CI: 1.041–5.487, *P* = 0.040). Our findings are consistent with the study by Ma *et al*^[4]^, which reported that the A allele of *STK11* rs12977689 was associated with an increased risk of CAD in T2DM patients, with an OR of 1.572 (95% CI: 1.039–2.376, *P* = 0.035). These findings indicate that T2DM patients with the A allele of *STK11* rs12977689 have a 1.6-fold higher risk of developing CAD than those with the C allele. Furthermore, this study also investigated the mechanisms by which the *STK11* gene influences CAD morbidity and found that the SNP rs12977689 was associated with BMI, a marker linked to disturbances in metabolic regulation. Disturbances at the cellular metabolic level may lead to adiponectin activation, which triggers AMPK phosphorylation by STK11 through the inactivation of acetyl-CoA carboxylase, resulting in fatty acid oxidation.

The male predominance in our sample is consistent with the higher prevalence of CAD in men, particularly within the age range of our study population^[9]^. Subgroup analysis (***Table 3***) using crude ORs revealed that the *STK11* rs12977689 polymorphism was associated with increased CAD risk in both males (OR = 2.33) and females (OR = 3.60), indicating a consistent trend across sexes. Although the ORs did not reach statistical significance because of the small sample sizes within the subgroups, the stronger crude association observed in females suggests potential sex-specific effects that warrant further investigation.

**Table 3:** Subgroup analysis of *STK11* rs12977689 C>A polymorphism and CAD risk in T2DM patients based on sex

Sex	Allele type	Case (*n*)	Control (*n*)	OR (CI)	*P*
Male	Polymorphism (CA/AA)	27	17	2.333 (0.957–5.689)	0.073
	Wild type (CC)	15	22		
Female	Polymorphism (CA/AA)	6	5	3.600 (0.464–27.936)	0.202
	Wild type (CC)	2	6		
Abbreviations: CAD, coronary artery disease; CI, confidence interval; OR, odds ratio; T2DM, type 2 diabetes mellitus.					

One study showed that 39.2% of T2DM patients were obese and 25.6% had hyperlipidemia. Notably, 84.1% of patients had LDL levels ≥ 70 mg/dL, while 56.5% had LDL levels ≥ 100 mg/dL. This study further demonstrated that higher HbA1c values were correlated with increased levels of LDL cholesterol and triglycerides (TG), consistent with previous studies that reported a correlation between poor glycemic control and elevated levels of total cholesterol, TG, and LDL cholesterol. These findings indicate that effective glycemic control is likely to lower LDL cholesterol and TG levels in diabetic patients^[10]^.

The mechanism by which smoking causes CAD is linked to an increase in systemic catecholamines, which impairs oxygen transport capacity and promotes atherosclerosis through endothelial dysfunction, oxidative stress, and thrombosis. Additionally, smoking raises the risk of other cardiovascular diseases, such as heart failure^[3,11]^.

Furthermore, the *STK11* rs12977689 SNP examined in the present study is located in the intron region. SNPs in the intron region are known to regulate gene expression and alter gene splicing, which affects AMPK activation related to the angiogenesis process in the myocardium. Therefore, the SNP *STK11* rs12977689 may serve as a genetic marker influencing the structure or catalytic activity of the STK11 enzyme^[4]^.

Our study demonstrated no significant differences between the two groups in terms of diabetes duration, sex, history of hypertension, smoking history, and blood cholesterol levels. This lack of significant difference may be attributed to the sample size or the specific demographic characteristics of the study population. Although the sample size was calculated to ensure sufficient statistical power, the single-center design and the relatively small sample size may limit the generalizability of the findings. Further studies with larger sample sizes are warranted to validate these results.

The present study identified a significant association between the *STK11* rs12977689 C>A polymorphism and CAD risk in T2DM patients. Our findings, with an odds ratio of 2.389, align with previous research; however, the magnitude of association observed in our Indonesian population appears slightly higher than that reported in previous studies, suggesting possible population-specific effects. Future studies involving larger, multi-center samples are needed to validate these results and further clarify potential ethnic or population-based differences in genetic susceptibility.

This study received no external funding.

Yours sincerely,Made Edwin Sridana^1,^^✉^, Wira Gotera^1^, Bagus Ari Pradnyana Dwi Sutanagera^2^, Made Ratna Saraswati^1^ ^1^Internal Medicine Department, Faculty of Medicine, Udayana University, Denpasar, Bali 80113Indonesia;^2^Cardiology Department, Faculty of Medicine, Universitas Udayana/Ngoerah Hospital, Denpasar, Bali 80113, Indonesia.^✉^Corresponding author: Made Edwin Sridana, E-mail:edwin@unud.ac.id, ORCID: 0000-0001-8670-0099.
